# Antiviral Prescriptions to U.S. Ambulatory Care Visits with a Diagnosis of Influenza before and after High Level of Adamantane Resistance 2005–06 Season

**DOI:** 10.1371/journal.pone.0008945

**Published:** 2010-01-28

**Authors:** Yu-Hsiang Hsieh, Kuan-Fu Chen, Charlotte A. Gaydos, Richard E. Rothman, Gabor D. Kelen

**Affiliations:** 1 Department of Emergency Medicine, Johns Hopkins University School of Medicine, Baltimore, Maryland, United States of America; 2 Department of Medicine, Johns Hopkins University School of Medicine, Baltimore, Maryland, United States of America; 3 Department of Emergency Medicine, Chang Gung Memorial Hospital, Taipei, Taiwan; Mount Sinai School of Medicine, United States of America

## Abstract

**Background:**

Rapid emergence of influenza A viruses resistance to anti-influenza drugs has been observed in the past five years. Our objective was to compare antiviral prescription patterns of ambulatory care providers to patients with a diagnosis of influenza before and after the 2005–2006 influenza season, which was temporally concordant with the emergence of adamantane resistance. We also determined providers' adherence to Centers for Disease Control and Prevention (CDC) 2006 interim treatment guidelines for influenza after the dissemination of guidelines.

**Methodology/Principal Findings:**

We conducted a multi-year cross-sectional analysis using 2002–2006 data from the national representative ambulatory care surveys, National Ambulatory Medical Care Survey and National Hospital Ambulatory Medical Care Survey. Our main outcome measure was prescription of any anti-influenza pharmaceutical medication, including amantadine, rimantadine, oseltamivir, and zanamivir. Analyses were performed using procedures taking into account the multi-stage survey design and weighted sampling probabilities of the data source. Overall, there were 941 visits to U.S. ambulatory care providers for which the diagnosis of influenza was made, representing 12,140,727 visits nationally. Antiviral drugs were prescribed in 21.7% of visits. Even though prescription rates were not significantly different by influenza season (2001–02: 26.4%; 2002–03: 11.2%; 2003–04: 16.5%; 2004–05: 18.0%; 2005–06: 35.8%; 2006–07: 46.5%, p = 0.061), significantly higher prescription rates were observed in the high adamantane resistance period (18.7% versus 37.0%, p = 0.023), and after the announcement of the 2006 guidelines (18.5% versus 38.8%, p = 0.032). Use of adamantanes decreased over time, in that they were commonly used during influenza seasons 2001–03 (60.1%), but used much less frequently during seasons 2003–05 (31.9%), and used rarely after high adamantane resistance emerged (2.2%) (p<0.001). Adherence to 2006 guidelines was 97.7%. After March 2006, no prescriptions for adamantanes were given to patients with a diagnosis of influenza.

**Conclusions/Significance:**

In this nationally representative study of U.S. ambulatory care visits, we found a complete absence of the use of adamantanes in all ambulatory care settings after March 2006, closely corresponding to release of the 2006 CDC interim guidelines. Adherence to such practice is an essential element for control and prevention of influenza, especially during the era of emergence of resistance to anti-viral drugs.

## Introduction

Each year, Americans make more than 20 million clinic visits per year for influenza. Of these, only 19% of visits receive antiviral prescription from their medical providers [1]. This low rate of antiviral treatment observed in practice may hamper public health efforts to minimize mortality, shorten the course of disease, and decrease transmission in the communities for the current novel influenza A (H1N1) [2] or future pandemics.

Rapid emergence of influenza A viruses resistance to anti-influenza drugs has been observed in the past five years. First, resistance of seasonal influenza A viruses to once first-line anti-influenza drugs, the adamantanes (amantadine and rimantadine) emerged in the United States, with resistance rates increasing from 14.5% during the 2004–2005 influenza season, to 92.3% during the 2005–2006 season [3]. In response, the US Centers for Disease Control and Prevention (CDC) recommended that adamantanes should not be used for treatment or prophylaxis of influenza on January 14, 2006 in the interim treatment guidelines [4]. Since that time, after only 3 influenza seasons, rates of oseltamivir-resistant to seasonal influenza A virus (H1N1) stains have risen from 12% to nearly 99% [5]. Accordingly, the CDC now recommends that when influenza A (H1N1) virus infection or exposure is suspected, zanamivir or a combination of oseltamivir and rimantadine are more appropriate options than oseltamivir alone [6]. Only a few months later in the early summer of 2009, the first influenza pandemic in more than 40 years has arrived [2]. The CDC recommends use of oseltamivir or zanamivir for the first line treatment and/or prevention of infections since the circulating strain is resistant to the adamantanes [7]. Few cases of oseltamivir-resistance strains of novel influenza A (H1N1) have been documented [8].

Timely and effective dissemination of these interim treatment guidelines for influenza from CDC to medical providers is crucial to prescribe appropriate antivirals for patients with confirmed or suspected influenza. In the past decade, CDC has disseminated treatment information (which could be a CDC Health Advisory, Health Alert, or Health Update) via CDC Health Alert Network (HAN) to state and local health officers, public information officers, epidemiologists, state laboratory directors, weapons of mass destruction coordinators, HAN coordinators, as well as public health associations and clinician organizations [9]. Few, if any studies, have examined U.S. medical providers' antiviral drug prescription usage patterns and trends for patients with a diagnosis of influenza, before and after the emergence of drug resistance. Notably, studies have been conducted evaluating provider adherence with CDC treatment guidelines for other infectious diseases, with rates varying significantly across syndromes, i.e. 30% for pelvic inflammatory disease [10] and upper respiratory infections [11], 50%–85% for acute epididymitis [12], and over 97% for chlamydia [13].

Utilizing the national representative ambulatory medical care surveys National Ambulatory Medical Care Survey (NAMCS) and the National Hospital Ambulatory Medical Care Survey (NHAMCS) [14] by National Center for Health Statistics, CDC, we compared antiviral prescription patterns of ambulatory medical care providers for patient visits with a diagnosis of influenza before and after the 2005–2006 influenza season, which was temporally concordant with the emergence of adamantane resistance. We also determined medical provider's adherence to CDC 2006 interim treatment guidelines for influenza after the dissemination of the CDC guidelines.

## Materials and Methods

We conducted a multi-year cross-sectional analysis using 2002–2006 data from three national representative ambulatory medical care surveys. The data from these three surveys include weighted samples of U.S. patient visits to non-federally employed office-based physicians from the NAMCS, U.S. emergency department (ED) visits from the ED component of the NHAMCS, and U.S. hospital outpatient visits, from the outpatient component of NHAMCS. NAMCS and NHAMCS data were collected by a standard survey form collected by physicians, office workers, or hospital staff. The multi-stage probability sampling scheme, the visit, and the reliability of coding and data entry for NAMCS and NHAMCS have been detailed elsewhere [15]. The NAMCS and NHAMCS data are publicly accessible and deidentified. Accordingly, the Johns Hopkins University Medicine institutional review board (IRB) deemed our study as nonhuman-subjects research, exempt from IRB review.

In this study, we employed the data collected by the NAMCS and NHAMCS, including demographic characteristics, diagnoses, and medications of the patient visits. Up to three diagnoses were recorded as free text and coded centrally by Constella Group, Inc. and subject to quality control procedures [16]. A visit with a diagnosis of influenza was defined as International Classification of Diseases, Ninth Revision, Clinical Modification (ICD-9-CM) codes of 487, 487.0, 487.1 or 487.8. All visits with a diagnosis of influenza during the study period were included for analysis. Six (in 2002) or eight (2003–2006) medications that were ordered or provided were collected and coded in the data set. Our main outcome measure was prescription of any anti-influenza pharmaceutical medication, including amantadine, rimantadine, oseltamivir, and zanamivir. A visit with a prescription of anti-influenza drug by providers was defined as medications matching for NAMCS and NHAMCS drug entry codes for amantadine (Symmetrel), rimantadine (Flumadine), oseltamivir (Tamiflu), and zanamivir (Relenza).

NAMCS and NHAMCS data from the years 2002–2006 were merged for data analysis. A sample weight that considers selection probability, nonresponse adjustment, and ratio adjustment for different total sample size each year is assigned for each patient visit to generate unbiased national estimates of ambulatory medical care visits. Although the sampling fraction is relatively small, the weighted numbers calculated by the method suggested by the CDC represent unbiased national estimates of the US ambulatory medical care population. Study period was categorized into 6 influenza seasons beginning October 1 to the end of September of the following year. In addition, study periods were further categorized into (1) pre-high adamantane resistance period versus high adamantane resistance period, based on the time when adamantane resistance rates reached over 90% [3], i.e. October 2005, or (2) before versus after the announcement of the 2006 interim treatment guidelines, based on the time of release of the 2006 CDC interim treatment guidelines [4], i.e. January 14, 2006. Adherence to the 2006 CDC interim treatment guidelines was defined as no adamantane prescribed to patients with a diagnosis of influenza, following the announcement of the treatment guidelines on January 14, 2006.

Descriptive demographic analyses of patient visits with a diagnosis of influenza, and patient visits with antiviral medications for influenza, were performed. Comparison of proportions, e.g. prescription rate in pre-high adamantane resistance period versus that in high adamantane resistance period, was assessed by chi-square test. All p values were 2-sided, with p<0.05 considered significant. Multivariate analyses and estimation of 95% CI were not conducted if the sample size of interest was less than 30, as the estimate is considered unreliable under NAMCS and NHAMCS analysis recommendations [17]. Analyses were performed using SAS 9.1 SURVEYFREQ procedure which takes into account the multi-stage survey design and weighted sampling probabilities of the data source (SAS version 9.1, SAS Institute Inc., Cary, North Carolina). Results were reported as weighted frequencies, percentages, and 95% confidence intervals (CI).

## Results

Overall, during 2002–2006, there were 941 visits to U.S. ambulatory care providers for which the diagnosis of influenza was made in the sample, representing 12,140,727 (95% CI: 10,116,136–14,165,318) patient visits nationally, or 0.22% (95% CI: 0.18–0.25) of all visits over this time. Demographics, type of ambulatory care, and influenza season in which patient visits resulted in a diagnosis of influenza were summarized in Table 1. Office-based physician visits represented the highest volume (9,459,918 visits, 77.9%), followed by ED visits (1,872,200 visits, 15.4%) and outpatient visits (808,609 visits, 6.7%). Among visits with a diagnosis of influenza, those which occurred during periods of high adamantane resistance, and after the announcement of 2006 CDC interim treatment guidelines accounted for 18.7% and 15.3% of all visits, respectively.

**Table 1:** National estimates of ambulatory medical care visits with a diagnosis of influenza and the antiviral prescription by demographics in the United States, 2002–2006.

Characteristics	% of National estimated visits (95% CI)		p-value
	Diagnosis of Influenza (N = 12,140,727)	Antiviral Prescription in Visits with Influenza in specific subgroup	
**Overall**	-	21.6 (16.7–26.6)	
**Age group, y**			0.225
0–9	37.9 (29.5–44.2)	19.3 (9.3–29.3)	
10–19	17.0 (13.1–21.0)	31.1 (17.1–45.1)	
20–29	9.1 (6.4–11.9)	19.2 (9.7–28.6)	
30–39	13.1 (8.5–17.8)	29.1 (13.3–44.8)	
40–49	10.4 (7.3–13.6)	21.0 (NA)	
50–59	3.8 (1.7–6.0)	9.1 (NA)	
≥60	9.5 (6.4–12.7)	11.8 (NA)	
**Sex**			0.548
Female	54.1 (47.8–60.4)	20.3 (13.6–27.0)	
Male	45.9 (39.6–52.2)	23.2 (16.4–30.1)	
**Race**			0.168
White	83.6 (76.9–90.3)	21.2 (16.5–25.9)	
Black	11.8 (5.2–18.3)	13.6 (2.6–24.6)	
Other	4.7 (2.0–7.3)	50.2 (NA)	
**Ethnicity** *			
Hispanic	11.6 (7.9–15.3)	21.9 (NA)	0.985
Non-Hispanic	83.8 (78.3–89.3)	21.8 (16.8–26.7)	
**Insurance Type**			
Private	61.2 (56.2–66.2)	18.8 (14.0–23.5)	0.052
Public	25.8 (20.8–30.8)	16.2 (7.6–24.8)	
Self-Pay	5.5 (2.9–8.1)	71.7 (NA)	
Other/Unknown	7.5 (4.5–10.5)	27.2 (NA)	
**Urban Status**			0.337
MSA†	85.1 (77.7–92.5)	20.4 (15.0–25.8)	
Non-MSA	14.9 (7.5–22.3)	28.6 (12.4–44.9)	
**US Region**			0.004
Northeast	18.6 (13.4–23.8)	12.3 (NA)	
Midwest	18.4 (16.2–24.2)	10.8 (5.2–16.4)	
South	43.2 (34.5–52.0)	31.0 (21.1–40.8)	
West	19.7 (14.5–25.0)	20.1 (9.7–30.6)	
**Ambulatory Care**			0.287
Emergency Department	15.4 (12.5–18.3)	29.0 (21.1–36.9)	
Outpatient	6.7 (3.2–10.1)	18.9 (7.2–30.7)	
Physician Office	77.9 (73.6–82.2)	20.4 (13.9–27.0)	

* Ethnicity missing for 4.7% of sample.

† MSA: Metropolitan Statistical Area.

‡ NA: Not applicable due to the sample size of interest in the surveys was less than 30, as the estimate is considered unreliable under NAMCS and NHAMCS analysis recommendations.

Antiviral drugs against influenza were prescribed in 21.7% of visits (weighted 2,629,129 visits; 95% CI: 1,886,292–3,371,966). Prescription rates by each demographic characteristic and type of ambulatory care were summarized in Table 1. Although EDs had the highest prescription rate, (29.0%), there was no statistical difference observed by type of ambulatory care setting (p>0.05). In addition, there were no statistical differences in prescription rates by demographic variables except by the U.S. region. Even though, prescription rates were not significantly different by influenza season, significantly higher antiviral drug prescription rates were observed in the high adamantane resistance period, and after the announcement of the 2006 CDC interim treatment guidelines (Table 2).

**Table 2:** National estimates of ambulatory medical care visits with a diagnosis of influenza and the antiviral prescription by influenza season, high adamantane resistance period, and announcement of 2006 CDC guidelines in the United States, 2002–2006.

Characteristics	% of National estimated visits (95% CI)		p-value
	Diagnosis of Influenza (N = 12,140,727)	Antiviral Prescription in Visits with Influenza in specific subgroup	
**Influenza Season**			0.061
2001–02 (Jan 2002–Sep 2002)	20.3 (11.1–29.4)	26.4 (10.4–42.3)	
2002–03 (Oct 2002–Sep 2003)	18.3 (10.7–26.0)	11.2 (NA)	
2003–04 (Oct 2003–Sep 2004)	24.3 (17.7–30.9)	16.5 (4.8–28.2)	
2004–05 (Oct 2004–Sep 2005)	18.4 (12.3–24.5)	18.0 (8.9–27.1)	
2005–06 (Oct 2005–Sep 2006)	16.5 (11.8–21.2)	35.8 (24.5–47.1)	
2006–07 (Oct 2006–Dec 2006)	2.2 (NA)	46.5 (NA)	
**High Adamantane Resistance Period**			0.023
No (Before October 2005)	81.3 (76.5–86.1)	18.1 (12.7–23.5)	
Yes (After October 2005)	18.7 (13.9–23.5)	37.0 (24.8–49.3)	
**2006 CDC Guidelines (Jan. 14 2006)**			0.032
Before Announcement	84.7 (80.0–89.3)	18.5 (13.4–23.7)	
After Announcement	15.3 (10.7–20.0)	38.8 (24.8–52.8)	

* NA: Not applicable due to the sample size of interest in the surveys was less than 30, as the estimate is considered unreliable under NAMCS and NHAMCS analysis recommendations.

**Figure 1:**
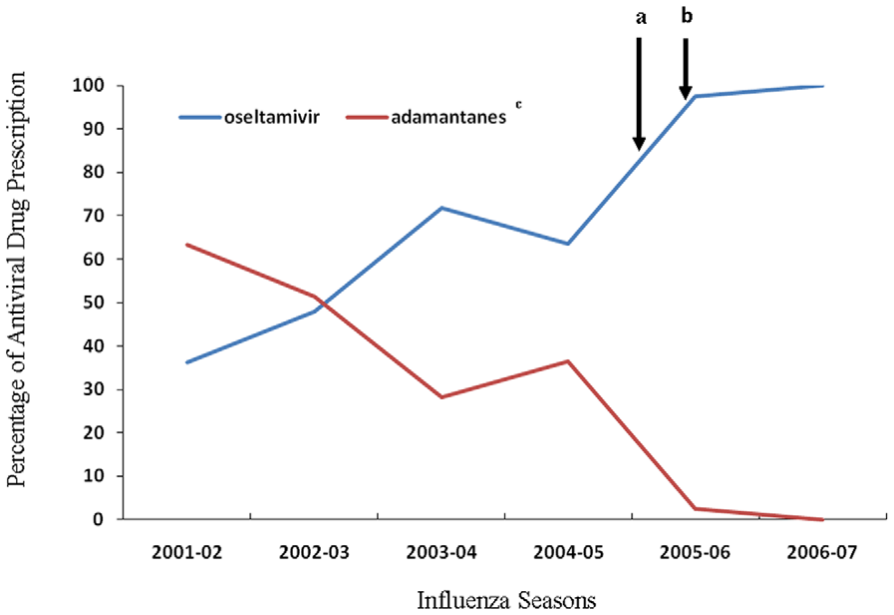
Prescription of anti-influenza agents for patient visits with a diagnosis of influenza in US ambulatory care including emergency department visits, outpatient visits and physician office visits, by antiviral drug, influenza season 2001–2007. **^a^** The time CDC declared interim guidelines for use of anti-influenza drugs. **^b^** The start of high adamantane resistance period. **^c^** Adamantanes include amantadine and rimantadine.

Numbers of antiviral prescriptions for influenza in U.S. ambulatory care visits resulting in a diagnosis of influenza were summarized in Table 3. Use of adamantanes decreased over time, in that they were commonly used during influenza seasons 2001–02 and 2002–03 (60.1%), but were used much less frequently during seasons 2003–04 and 2004–05 (31.9%), and were used rarely after high adamantane resistance emerged (2.2%) (p<0.001) (Figure 1). In contrast, oseltamivir accounted for only 36% of total antiviral prescription during the influenza season 2001–02, but increased over time, becoming the predominant anti-influenza prescription in all three ambulatory care settings during the high adamantane resistance period, i.e. after October 2005 (97.8%; 95% CI: 93.9–100%). There were no statistical differences in anti-influenza prescriptions by type of ambulatory care or by other demographic variables, before or during high adamantane resistance period (data not shown). Therefore, a multivariate analysis was not performed on use of adamantanes or oseltamivir and time.

**Table 3:** Numbers of antiviral prescription for influenza in U.S. ambulatory care visits with a diagnosis of influenza during 2002–2006.

Study Periods	Visits (%) with Antiviral Prescription (N = 2,629,129)	No.(%) Prescription of Adamantanes in Each Period	No. (%) Prescription of Oseltamivir in Each Period	p-value
**Influenza Seasons**				0.0020†
2001–02 (Jan 2002–Sep 2002)	648,207 (20.3%)	411,447 (63.5%)	235,567 (36.3%)*	
2002–03 (Oct 2002–Sep 2003)	249,842 (18.3%)	128,230 (51.3%)	119,928 (48.0%)*	
2003–04 (Oct 2003–Sep 2004)	487,631 (24.3%)	137,420 (28.2%)	350,211 (71.8%)	
2004–05 (Oct 2004–Sep 2005)	402,208 (18.4%)	146,331 (36.4%)	255,877 (63.6%)	
2005–06 (Oct 2005–Sep 2006)	718,061 (16.5%)	18,843 (2.6%)	699,218 (97.4%)	
2006–07 (Oct 2006–Dec 2006)	123,180 (2.2%)	0 (0%)	123,180 (100%)	
**High Adamantane Resistance Period**				0.0001
No (Before October 2005)	1,787,888 (81.3%)	823,428 (46.1%)	961,583 (53.8%)*	
Yes (After October 2005)	841,241 (18.7%)	18,843 (2.2%)	822,398 (97.8%)	
**2006 CDC Guidelines (Jan. 14 2006)**				0.0008
Before Announcement	1,906,290 (84.7%)	825,605 (43.3%)	1,077,808 (56.5%)*	
After Announcement	722,839 (15.3%)	16,666 (2.3%)	706,173 (97.7%)	

* Zanamivir was prescribed, therefore, percentages of prescription in these horizontal rows were not added up to 100%.

† P-value cannot be computed for all influenza seasons because at least one table cell has 0 frequency (season 2006–07). P-value was calculated based on the comparison of influenza season 2001–02 to season 2005–06.

Overall, adherence with the 2006 CDC interim treatment guidelines was 97.7% (95% CI: 93.0%–100%). After March 2006, no prescriptions for adamantanes were given to ambulatory-care patients with a diagnosis of influenza (last prescription in the dataset: office-based physician visit: February 2005; ED visit: February 2006; outpatient visit: March 2006).

## Discussion

In this nationally representative study, we found that the use of adamantanes markedly decreased after the influenza 2002–2003 season, and were rapidly replaced by oseltamivir as the predominantly prescribed anti-influenza drug therapy in U.S. non-federally employed office-based physician, ED, and outpatient visits. The major shift occurred during the early influenza 2005–2006 season, paralleling the rapid emergence of adamantane resistance of influenza A viruses seen in both Asia [18] and the U.S. [3]. We also identified a complete absence of adamantane prescriptions in all ambulatory medical care settings after March 2006, which closely corresponded to release of the CDC interim guidelines for use of antiviral agents for 2005–06 influenza season. Both findings suggest that the antiviral prescribing practice for influenza treatment or prophylaxis among U.S. ambulatory medical care providers was closely in line with the most up-to-date global epidemiological resistance patterns, as well as the CDC recommendations, which provides encouraging news regarding medical provider's capacity to adhere with evolving changes in antiviral treatment recommendations, particularly relevavant to the current circulating novel H1N1 influenza virus, as well as other potential emerging influenza pandemics.

Prescription patterns and compliance with existing CDC treatment guidelines vary by diseases. The reasons for the variation are beyond the scope of our study. However, potential contributors include severity of disease, individual provider perception regarding the individual patient and public health impact of effective treatment/post-exposure prophylaxis, the intensity of media reporting, and the ease of implementation. Together these factors could contribute to the significant differences between the nearly perfect provider adherence observed for CDC treatment guidelines for influenza, versus that seen for PID, upper respiratory infections, and acute epididymitis [10], [11], [12]. One infectious disease in which clinician adherence with CDC guidelines is also high is genital chlamydial infection, where, effective dissemination of updates, implementation of electronic order-entry systems, and national continuing medical education efforts have been reported to increase rates of compliance in at least 2 large managed care organizations [13].

Since patients with suspected influenza visit a variety of ambulatory care settings in the U.S., timely and concentrated electronic communication messages and interaction between the CDC and each medical society and institution, as well as effective dissemination from the society or institution to its own members is likely critical for ensuring adherence with the most up to date treatment guidelines during a pandemic. Individual institutional utilization of modern information technology, e.g. electronic medical record system, is also certainly important in promotion of new treatment guidelines and will aid in minimizing inappropriate use of treatment regimen in the case that guidelines are modified [19], [20]. To what extent these interventions contributed to the high rates of compliance seen here is not known but warrants further study.

The NAMCS and NHMACS databases do not have a specific focus on adamantanes prescriptions and potential emergence of resistance, as this is not the primary purpose of these survey tools. Accordingly, associated factors not collected in NAMCS and NHAMCS, such as virus subtype coverage (i.e., influenza A versus B), medication side effects, prior antiviral prescriptions, and clinician's knowledge of emerging resistance, may have contributed to the observed trends. In addition, other factors, e.g. ease of antiviral medication administration, need to adjust dosage according to age and renal function, and prior antiviral prescriptions may have contributed to the observed temporal decline from 2002–2005 in prescription of adamantanes in U.S. ambulatory care settings. Finally, ICD-9-CM coding, which is not a gold standard for influenza case reporting, was used to define cases of influenza rather than other potentially more valid standards – e.g. laboratory confirmed tests.

In conclusion, our study demonstrated extremely high adherence to the most current national treatment recommendations for influenza among non-federal-employed ambulatory medical care providers in the U.S. Adherence to such practice is an essential element for control and prevention of influenza, especially during the era of emergence of resistance to anti-viral drugs.
